# Barbara Natterson-Horowitz and Kathryn Bowers, *Wildhood: The Epic Journey from Adolescence to Adulthood in Humans and Other Animals*

**DOI:** 10.1093/emph/eoaa001

**Published:** 2020-06-18

**Authors:** Carlo C Maley

**Affiliations:** Arizona Cancer Evolution Center Biodesign Institute, Arizona State University 1001 S. McAllister Ave, Tempe, AZ 85287, USA

Wildhood is a masterful exploration, across species, of the period of development between childhood and adulthood. There are consistent challenges in that period, faced by most organisms, including learning how to be safe, negotiating social status hierarchies, learning how to court mates, and learning how to acquire food and other necessities for survival. The solutions that evolution has found for overcoming those challenges are surprisingly consistent across the animal kingdom, including humans. The lessons of the book come in the form of stories of individual animals whose trials and tribulations have been documented by scientists. The book helps us understand that the sometimes bewildering behaviors of adolescents are actually rational responses to the challenges they are facing. The book gives us insights into the underlying biology driving those behaviors. It may also open your eyes to a new understanding of your own development.

One of the most profound and disturbing insights of evolutionary biology is that humans are animals that we are a branch of the tree of life and not above it. One of the most profound and distressing periods of life is the long transition from childhood to adulthood. Adolescence collides spectacularly with the tree of life in *Wildhood: The Epic Journey from Adolescence to Adulthood in Humans and Other Animals*, by Barbara Natterson-Horowitz and Kathryn Bowers.

Wildhood divides the transformation from childhood to adulthood into four stages defined by the challenges they pose: learning to be safe, negotiating status hierarchies, learning to court mates and becoming self-reliant. This period actually extends past what we typically call adolescence, as full maturity takes time. The human brain does not finish developing until our early 30’s. So the authors introduce the term ‘wildhood’ to capture this extended period of development from puberty to mature adulthood.

Because, we have all gone through (or are going through) those harrowing years, this book is likely to bring back memories, and let you see them in a new light. If you are a teen or pre-teen, or are currently caring for one, this book is for you. If you have ever shaken your head at the crazy s#!t that adolescents do, this book is also for you. It turns out that adolescents all across the animal kingdom do the same things, and for good evolutionary reasons.

Why would you choose to swim into great white shark-infested waters, especially if you are one of their favorite meals? But adolescent sea otters do that all the time: willingly approach their predators, usually in groups, as part of what is called predator inspection. It is a great way to gain experience, experience that is hard to gain any other way, while minimizing the risk. Predator inspection has been documented in fish, birds and across mammals. Both experiments and observations show that the experience gained from predator inspection provides a significant survival advantage over predator-naive individuals. This advantage appears to be sufficient to compensate for the eventuality that the predator often kills a few of its inspectors. However, taking these risks in groups reduces the risk to any one individual. It is probably no accident that humans take more risks as teenagers than at other stages of life, and that they tend to do this in groups. Most of us do not live in predator rich environments anymore, but I have to agree with the authors that the popularity of horror movies, especially among teenagers, is likely the modern equivalent of predator inspection. And the propensity of human teenagers to take risks in groups is not in question. The time I remember clearly thinking ‘this is f#%king crazy’ was speeding down a strip of road near the mall, in a car full of fellow teenagers, in a spontaneous drag race with another car full of our friends. It is also likely there was a fair dose of status hierarchy negotiation going on at the same time.

Speaking of social hierarchies, hyenas are the worst kind of helicopter parents. High-status hyenas guide their young in who to pick on, to start asserting the youngster’s status. They constantly intervene in the social negotiations of their offspring, assuring their success, like parents of the privileged calling up their children’s professors to intercede. This leads to non-genetic, cultural inheritance of status. In many species, social status does not have to be earned by the individual through one form of competition or another. It is simply bequeathed (and reinforced) by the parents. As the authors say, ‘level playing fields do not exist in nature’. That is not to say that the hierarchies are entirely fixed. There is some wiggle room, and individuals can gain or lose status during their lives. Particularly in humans, during wildhood, we can choose to join groups where status is largely based on competence for a skill of our choice. Communities organized around a common interest, such as dance, theater, music, sports, debate, etc., go a long way to mitigate the injustice and burden of inherited social status (though this does not always stop stage parents from trying to tip the scales).

Threats to social status can be buffered by the coalitions that animals start building in their wildhood. The sorting of animals into social hierarchies during wildhood can have long-lasting effects on their behavior. From the fifth grade on, I spent most of my social time with friends a year older than me, or with my older brother and his friends. It was not until senior year after my older friends had graduated that I fully stepped into leadership roles. I suspect that those formative experiences, both in lower status and higher status roles, might be part of why I am happy and comfortable acting the role of the right-hand man, but also enjoy building and leading coalitions.

Wildhood is also an interdisciplinary study, and the alchemy of mixing animal behavior studies with psychiatry has produced gold. We tend to think of serotonin as a hormone that regulates mood, ever since the success of selective serotonin reuptake inhibitors in treating depression. Natterson-Horowitz and Bowers show that a more useful hypothesis is that serotonin is part of the reward system for changes in social status. Because those changes are so important to fitness, we, and many other animals, have evolved to experience severe pain and depression with the loss of status, and elation with the gain of status. In fact, evolution has re-used the same pain circuitry that evolved for physical pain to provide feedback for social pain. One consequence of this is that social pain can be alleviated with Tylenol. Studies of fish, mammals and even invertebrates show that serotonin regulates status hierarchies. Give a subordinate crayfish a dose of serotonin and it will stop withdrawing from fights and start displaying dominance behaviors that force others to back down, which in the end will actually enhance their status. These serotonin-based status hierarchies all come online during adolescence, shaping our neurochemistry through these formative social experiences.

This insight has changed how I understand my own high school days. Up until junior year, I was getting mixed signals of my status. I was in a coalition of older boys, but I often organized our activities. I was rising to eventually captain of our varsity soccer team, but our team sucked. I was successful in school and had a good coalition of friends, but I could not convince a girl to date me. They all just wanted to be friends. That was especially painful. I am that sure my serotonin levels were declining until I lingered at school 1 day at the end of a rehearsal. I had been cast as the romantic lead in the (rock) musical, my junior year. My best friend, that alpha-boy who would not initiate get-togethers, had a supporting role in the musical. As I watched him rehearse his one song, I had a sudden shift in perspective. He looked like a hero up on stage, a protagonist, a celebrity, and it struck me—*that* is how other people are seeing *me* when I am up on stage. At the time, it felt as if I had just decided to be confident, and I was. It was a wonderful feeling, as all that serotonin came flooding back. A few days later, the girl I had been pursuing for months agreed to be my first girlfriend.

Before reading this book, I remembered wanting to have sex when I was 12 years old and thought that maybe our culture has it wrong when we say early teenagers are not ready. I thought it was just the risk of pregnancy and the emotional hooks that come with sex that make teenagers not ready. But the section of the book on courtship changed my mind. It turns out, strangely, that many species delay mating for years after they become physically able to have sex. They do not even try. This is a period of intense learning—not learning how to perform the act of mating, but learning how to court a mate, how to negotiate desire, inhibition and the rituals that amount to choice and consent in mating. At 12 years, I was definitely not ready to negotiate that. At 50 years, I am still not sure that I am.

The authors argue, quite rightly, that our society makes the mistake of focusing sex education on the mechanics of sexual reproduction but does very little in the way of formal education about courtship, sending and interpreting signals, and consent. While the authors’ systematic survey and phylogenetic analysis showed that coercive sex is common across animal species, consensual sex is *more* common. Most animals learn courtship and mating behaviors through observation of their elders. It is problematic that today many (most?) human adolescents are learning about mating behaviors through pornography.

The story of courtship in wildhood is told through the story of Salt, the first humpback whale (that we know of) to be given a name. She was identified and named off of Cape Cod in the 1970’s and has been observed there seasonally ever since, along with her children, her grandchildren and even her great-grandchildren. Because of her notoriety, we chose her as the donor when we set out to sequence the genome of the humpback whale that we published recently [1]. It was fun to hear more of her story, and the courtship rituals of humpbacks in Wildhood.

For those parents who worry about boomerang children, and for their children who might feel some shame that they are still living at home in their 20’s and beyond, the authors provide reassurance. In many species, physically mature children often delay dispersal from their parents, thereby gain both additional resources and skills that serve them well when they do eventually leave. Those ‘helpers in the nest’ that stay behind to help parents raise their younger siblings are often better parents when their time comes. It also puts them in the right place, at the right time, if their parents die, so that they can inherit their parent’s territory. This reminded me of a recent family dinner when our teenage daughter inquired ‘When you die, can I have this house?’ At least she is not swimming in shark-infested waters or drag racing at the mall (to our knowledge).

Many of the behaviors in the book will not be a surprise to experts in animal behavior, but for students of human behavior, there are a pile of gifts wrapped in this book. The best insights come from the interdisciplinary fusions of behavioral ecology, psychiatry, collective behavior, evolutionary biology and phylogenetic analyses. The methods that the authors used are also interdisciplinary, including systematic reviews common in medicine, phylogenetic analyses from evolution and comparative biology, and field studies from animal behavior. The book is extensively researched and documented in voluminous endnotes. The rigor of these methods can be easily missed, in part, because there are so few graphs and only one small phylogeny presented in the book. The narratives are front and center. That is understandable, given that the book is targeting a popular audience, but I would have loved to have geeked out over phylogenies showing the patterns of these wildhood behaviors across the tree of life. It was not always clear to me whether a behavior was an ancient ancestral trait or had emerged many times through convergent evolution. However, the overall message is clear. The challenges of wildhood, and the behaviors that have evolved to meet them, are near universal in animals, and humans are no exception.

Could Wildhood be used to train students? There are a large number of concepts covered in each of the four sections on predator avoidance, social hierarchies, courtship and food acquisition. Each subsection, covering an idea, is typically a few pages long, and is illustrated with examples, often multiple examples. But underneath those examples are the methods and fundamental tools that are used in evolutionary biology. My colleagues and I tried to map out the mental tools that distinguish an excellent from an untrained evolutionary biologist, so as to focus our training of students on developing those mental tools. Our answer, most broadly, was that excellent evolutionary biologists use population thinking, tree thinking, careful observation and a compendium of compelling examples from nature. Wildhood embodies all four of these mental tools. The puzzling and painful behaviors of wildhood are generally, ultimately fitness-enhancing, and thus spread in populations through natural selection. Most of those behaviors either evolved independently multiple times across the animal phylogeny or derive from common ancestors who had already evolved those behaviors. The driving narratives of the book are the compelling examples of individual species, and often the stories of individuals of those species, going through the familiar growing pains of adolescence. These observations come from the careful studies of the authors and many of their/our colleagues that they visited and interviewed. To be fair, the emphasis of the book is on the examples and observations, not explicit training in phylogenetics, systematic reviews or adaptationist thinking, but those methods are present throughout the book.

I expect Wildhood to be a compelling medium for educating adolescents in evolutionary biology, animal behavior, evolutionary psychology and a support for their mental health because it addresses their greatest concerns at their current time of life. It pulls back the curtain on why they are feeling this way and why their peers are behaving in the ways they do. It provides the comfort of showing them that they are not alone—not only in the fact that all other human adolescents are facing the same challenges and pain but also that virtually all animals are facing the same challenges and pain. It does not explicitly teach them population or tree thinking, but it provides them, over and over again, fascinating examples from the natural world that they can relate to. It also obliterates the siren song of human exceptionalism, which sets them up well for both understanding humans and animals. Wildhood could be valuable in both high school and college curricula. There is a section of the wildhood.com web site devoted to teaching materials, but it has not been populated yet.

There are so many nuggets and insights in the book that I do not have time to go into (e.g. humans mainly use words for grooming—I had never thought of that). I have only given you a taste of those that resonated most with my own wildhood. Reading this book is enlightening, both scientifically and personally.
